# Vascular Access Management in Patients on Catheter-Based Hemodialysis

**DOI:** 10.3390/jcm15072714

**Published:** 2026-04-03

**Authors:** Markus Plimon, Maria-Elisabeth Leinweber, Amun Hofmann, Fadi Taher, Johannes Werzowa, Marcus Säemann, Afshin Assadian

**Affiliations:** 1 Department of Vascular Surgery, Clinic Ottakring, Montleartstraße 37, 1160 Vienna, Austria; 2 Department of Medicine I, Hanusch Clinic, Heinrich-Collin-Straße 30, 1140 Vienna, Austria; johannes.werzowa@oegk.at; 3 Department of Medicine VI, Clinic Ottakring, Montleartstraße 37, 1160 Vienna, Austria

**Keywords:** vascular access, quality of care, resource management, hemodialysis, vascular surgery, end-stage renal disease

## Abstract

**Background/Objectives**: Hemodialysis (HD) is a life-sustaining treatment for an increasing number of patients around the globe. The options for vascular access (VA) in HD are arteriovenous fistulas (AVFs), arteriovenous grafts (AVGs), and central venous catheters (CVCs). AVFs are historically associated with better long-term outcomes. Recently, international guidelines have shifted to a more individualized approach driven by evidence that patient-specific factors influence the success of AVF creation. The initial “Vienna ACTS NOW” study revealed significant inter-center variability in CVC prevalence in Vienna. This two-year follow-up aimed to document the continuing variability in CVC-based HD management and to monitor the clinical outcomes and cost associated with an attempted conversion to AVF/G. **Methods**: This multi-center cohort study collected data (March 2023 to March 2025) on 153 CVC-based HD patients from six Viennese institutions. Primary endpoints included VA-related events and patency; the secondary endpoint was overall mortality. Costs were calculated using the Austrian Catalogue for Medical Services (MEL). **Results**: Overall, 28 (18.3%) out of 153 patients underwent AVF/G surgery, and 20 (71.4%) achieved successful cannulation. A total of 12 surgical and 14 endovascular interventions were performed to either support VA maturation or maintain patency. The median hospital admission was 3 days for VA creation and 4 days for later interventions. VA creation in patients that did not require later interventions cost 3130.44 € per patient, and it cost 11,893.02 € in patients that did. The proportion of AVF/G creation attempts varied from 0% to 40.9% between centers. Patients who underwent VA-creating surgery had a better rate of survival after two years compared to patients who did not undergo VA-creating surgery. (86.2% vs. 63.3% *p* < 0.02). Overall, 6.3% of deaths were related to VA management. **Conclusions**: Patient-specific characteristics and the capability of the healthcare system to timely detect and treat CKD might influence the outcome of patients. The proportion of CVC- and AVF/G-based HD might therefore be associated with the level of access patients have to the healthcare system and the efficiency of the care network. Our own data point towards a difference in CVC use between different centers in Vienna, not solely driven by patient characteristics, but by locally available resources and differences in policies.

## 1. Introduction

Hemodialysis (HD) is a life-sustaining treatment for an increasing number of patients around the globe. It is associated with high morbidity, high mortality, and a significant economic burden to healthcare systems. It is the most common form of chronic kidney replacement therapy (KRT), the others being kidney transplantation (KTX) and peritoneal dialysis (PD). Chronic kidney disease (CKD) incidence has more than doubled globally in the past 30 years, with a notable rise in KRT among elderly patients [1,2]. In Austria, the rise in KRT has largely been driven by the growing prevalence of type 2 diabetes and arterial hypertension in an aging population, which are key risk factors for the progression of kidney disease [3]. Optimizing end-stage CKD care, therefore, remains a relevant target from both clinical and public health perspectives. This includes vascular access (VA) care, where arteriovenous fistulas (AVFs) and grafts (AVGs) are routinely preferred over central venous catheters (CVCs). Numerous studies have shown that AVFs are associated with better long-term survival outcomes compared to AVGs and CVCs, with fewer reported complications such as infections and thromboses [4].

The historic Fistula First Breakthrough Initiative was initiated in the United States in 2003 to address the high proportion of CVC-based HD and increase the proportion of AVFs used for initiation and continuation of HD [5]. In recent years, however, international guidelines have shifted to a more individualized approach and towards the creation of a personalized end-stage renal disease life plan [6]. This change has been driven by emerging evidence that factors such as advanced age, diabetes, peripheral arterial disease, and frailty can influence the success of AVF creation and maintenance. In some patients, these factors can lead to higher failure rates and necessitate recurrent interventions, which may ultimately outweigh the benefits of a strict AVF-first policy. Consequently, VA management has become an area of considerable interest and variability, reflecting the challenges different healthcare systems face in optimizing care for dialysis patients [7].

Internationally, there is a wide variety of VA creation policies, reflecting region-specific challenges as well as cultural differences in providing healthcare. These differences in local policies, as well as a lack of coordination between centers, can lead to significant variability in VA use between centers situated in the same region. The impact of this effect on VA management can only be estimated using observational data since there is a lack of randomized trials or meta-analyses investigating this effect. Registry data points to a significant influence of center-level effects on the variation in AV access use across centers [8].

The “Vienna ACTS NOW—Vienna Vascular Access Studies on Exchange of Dialysis Catheters for Fistulas” study was initiated in 2023 to investigate the inter-center variability in VA management in Vienna. The study showed an inter-center variability in CVC proportions ranging from 27.6% to 94.4%, with a mean CVC proportion of 42.5%. Furthermore, there was a difference in CVC proportions in centers with an in-house vascular surgery service (34.1%) and in centers without an in-house vascular surgery service (63.8%) [9].

The data collected over two years in this study were used to document the possible continued difference in VA management of patients on CVC-based HD in Vienna. We aimed to document the clinical course of patients on continued CVC-based HD and to monitor the outcomes of patients who underwent AVF or AVG creation.

## 2. Materials and Methods

Vienna ACTS NOW was a multi-center, cross-sectional analysis investigating patients with CVC-based HD at 7 institutions providing chronic HD in Vienna at the cut-off date of 1 March 2023 [9].

All patients over the age of 18 receiving CVC-based HD at any of the participating centers on the cut-off date were eligible to participate in the study. The follow-up data collection period of the Vienna ACTS NOW cohort spanned from 1 March 2023 to 1 March 2025. The study utilized a standardized case report form (CRF, ePRO software Version 2022.3 by Castor, Amsterdam, The Netherlands) to collect data on VA history, complications, patient morbidity, and mortality in HD patients.

### 2.1. Definitions and Data

The primary study endpoint was the occurrence of VA-related events, including complications such as CVC-related infections and thrombosis, as well as patency rates following VA creation. The secondary endpoint was overall mortality, categorized by cause of death (e.g., cardiovascular, infectious, malignancy, access-related, etc.).

VA creation was categorized as either “forearm fistula”, “upper arm fistula”, “forearm graft”, “upper arm graft” or “other”. Revisions were recorded as either “endovascular” or “surgical”. An AVF/G was defined as “successfully punctured” after three consecutive AVF/G-based HD sessions.

The attending nephrologists recorded central-line-associated bloodstream infections (CLABSIs) and catheter-related bloodstream infections (CRBSIs). These were combined into an “infectious” category. CVC dysfunction because of thrombosis or material failure was combined into a “dysfunction” category. All other causes for CVC changes were grouped as “other”.

The causes of death were grouped into the categories “cardiovascular”, “malignancy”, “infectious”, “other” and “access-related” by the treating physicians. For patients for whom mortality information could not be retrieved, the cause of death was labeled “unclear”. All reported causes of death were reviewed by the study team and cross-checked with the report of the medical examiner.

All AVF/G-creating procedures during this study were performed by vascular surgeons. The AVF/G-maintaining procedures were performed either by vascular surgeons or interventional radiologists. The selection criteria for patients to undergo the creation or maintenance procedure of an AVF/G were left to the discretion of the treating physicians and local policies.

The cost of each procedure was calculated using the Austrian Catalogue for Medical Services (Medizinische Einzelleistungen—MEL). The MEL regulates the payout to hospitals in Austria as a function of the procedure performed and the length of admission.

To ensure data reliability, the study team performed plausibility checks on the data sets that were transmitted by the participating institutions. If data were implausible or incomplete, queries were sent to the respective institution for clarification.

### 2.2. Statistical Analysis

Analyses were performed using R v.4.4.3 (R Foundation for Statistical Computing, Vienna, Austria) in RStudio 2025.09.0 Build 387 (Posit Software, Boston, MA, USA).

Descriptive analyses were performed to represent patient characteristics. Normality was tested using Q-Q Plots and Shapiro–Wilk tests. The Mann–Whitney U Test was used to test for a statistical difference between medians, where appropriate. Person’s Chi-squared test with Yates’ continuity correction was used to determine if there was a significant association between categorical variables. The survival probability of patients was calculated using the Kaplan–Meier method, and comparisons between groups were made using the log-rank test.

### 2.3. IRB Approval

This study was authorized by the Ethics Committee of the City of Vienna, Austria (approval no. EK-22-104-VK, 28 November 2022, Amendment on 29 December 2024), and adhered to the principles laid out by the Declaration of Helsinki. All participants were provided with detailed study information, and written informed consent was obtained prior to enrollment in the Vienna ACTS NOW Study.

## 3. Results

### 3.1. Study Population

A total of 191 patients were included in the initial study. All the included patients received their CVC-based HD via a tunneled CVC [9]. We were able to collect the follow-up data on 153 (80.1%) patients treated at six of the seven initially participating institutions, with center number 3 failing to submit follow-up data.

**Figure 1:**
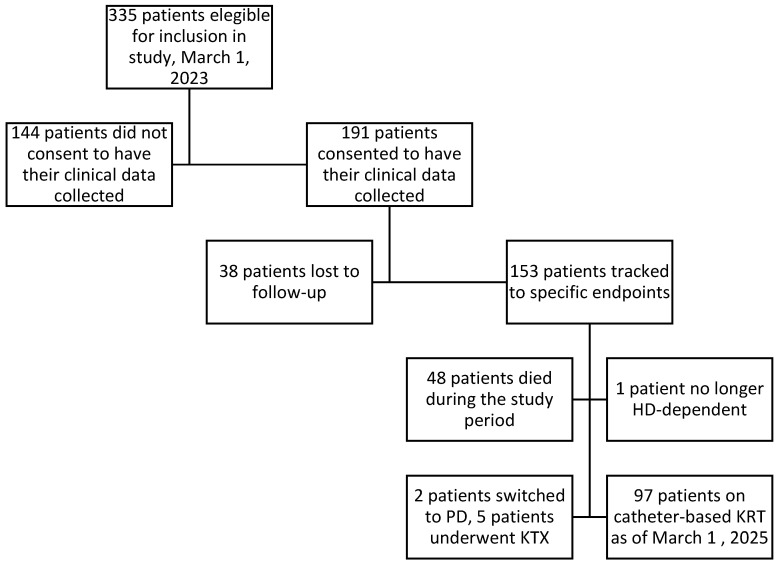
Patient recruitment and status at the end of the study.

At the onset of the study on 1 March 2023, 335 patients in the participating centers received CVC-based HD in Vienna. A total of 191 patients provided consent for the collection of their clinical data. By the study’s cut-off date, 1 March 2025, data were available for 153 patients, with 38 patients lost to follow-up. A workflow of the study and patient status is described in Figure 1.

### 3.2. Conversion to Arteriovenous Access

A total of 28 patients (18.3%) underwent the creation of an AVF or AVG. Overall, 27 patients (96.4%) had an upper extremity AVF created as their initial VA. Of the patients who underwent AVF/G creation, 20 (71.4%) achieved successful cannulation. For the initial VA creation, patients spent a total of 99 hospital days, with a median of 3 days per patient (Q1 2 days, Q3 4 days).

A total of 14 patients could be cannulated after spontaneous maturation. Six patients required either surgical or endovascular intervention to undergo successful cannulation. Seven patients required either surgical or endovascular intervention to maintain an AVF/G under use. These patients collectively underwent 24.96 patient-years of HD with AVF/G access. Two patients transitioned to PD, and five patients underwent KTX during the study period. Patient characteristics are described in Table 1.

Several reasons for failure-to-cannulate were observed: In two patients, the created access failed to mature and was abandoned, whereas in another patient, the access failed to mature, and the patient received a KTX shortly afterwards. Two patients did not consent to the puncture of the AVF; one no longer required KRT; one patient did not consent to further surgeries after their fistula failed to mature; and for another patient, the reason for the failure to cannulate was unclear.

A total of 18 patients underwent 12 surgical and 14 endovascular interventions during the study period to either support VA maturation or address the problem of an existing VA. Additionally, two patients received upper extremity grafts, and one patient underwent the creation of an upper extremity AVF following the failure of their initial AVF/G. For these revision procedures, patients spent a total of 126 hospital days, with a median of 4 days per patient (Q1 2 days, Q3 11 days).

Patients who consented to VA mapping during the study were more likely to undergo surgery than patients who did not consent to VA mapping, with an odds ratio of 3.93 (CI 1.55–10.48, *p* = 0.002).

The proportion of attempted AVF/G creation in the study population varied between centers, with a maximum of 40.9% and a minimum of 0% of patients undergoing surgery, as shown in Table 2.

During the follow-up period, a total of 153 patients accumulated 225.3 patient-years of CVC-based HD. Overall, 34 patients required a total of 39 CVC switches, with the majority (64.1%) attributed to CVC-related infections. As a result of CVC-related complications, patients spent a cumulative total of 358 days hospitalized. For those admitted to a hospital ward, the median length of stay was 6.5 days (Q1 2.25, Q3 15.0).

The rate of hospitalization due to CVC-related complications was 1.59 days per year of CVC-based HD. Specifically, the rate of hospitalization related to infectious CVC complications was 1.14 days per year of CVC-based HD.

### 3.3. VA-Associated Reimbursement

Ten patients were able to convert to AVF/G-based HD after successful AVF/G creation and maturation and did not require secondary interventions to ensure patency, with a mean and median procedure-related reimbursement of 3130.44 and 3085 € per patient. A total of 18 patients required either surgical or interventional revision to either achieve or maintain patency, with a combined mean and median reimbursement of their initial operation and revision of 11,893.02 and 10,390.5 € per patient. The total reimbursement of interventions for creating and maintaining vascular access and admissions in 28 patients was 245,378.81 € for the two-year follow-up period.

Overall, 22 patients required 25 CVC changes attributed to infectious complications with a mean and median reimbursement of 8711.72 and 7325 € per patient. A total of 12 patients underwent 14 CVC changes attributed to either dysfunction or other causes, with a mean and median procedure-related reimbursement of 6794.77 and 4508 € per patient.

**Figure 2:**
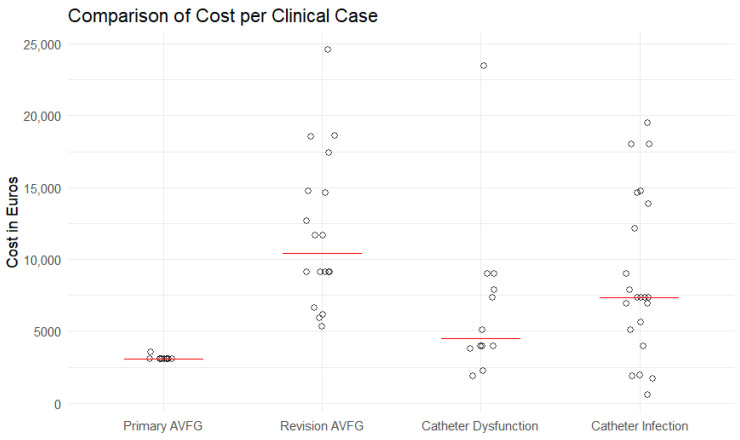
MEL-based reimbursement in Euros. Jitterplot with median displayed as the red line.

The total reimbursement for CVC-associated interventions and admissions in 34 patients was 273,194.98 € during the two-year follow-up period. The total cost of VA-associated interventions and admissions over the study period was 518,573.79 €, and the cost per patient year was 2072.14 €. The reimbursement of performed interventions is shown in Figure 2.

There was no significant difference in the 1- and 2-year survival probability of patients who underwent VA mapping during the study and those who did not (*p* = 0.7).

The 1- and 2-year survival probabilities of patients were 84.0% and 67.7%.

**Figure 3:**
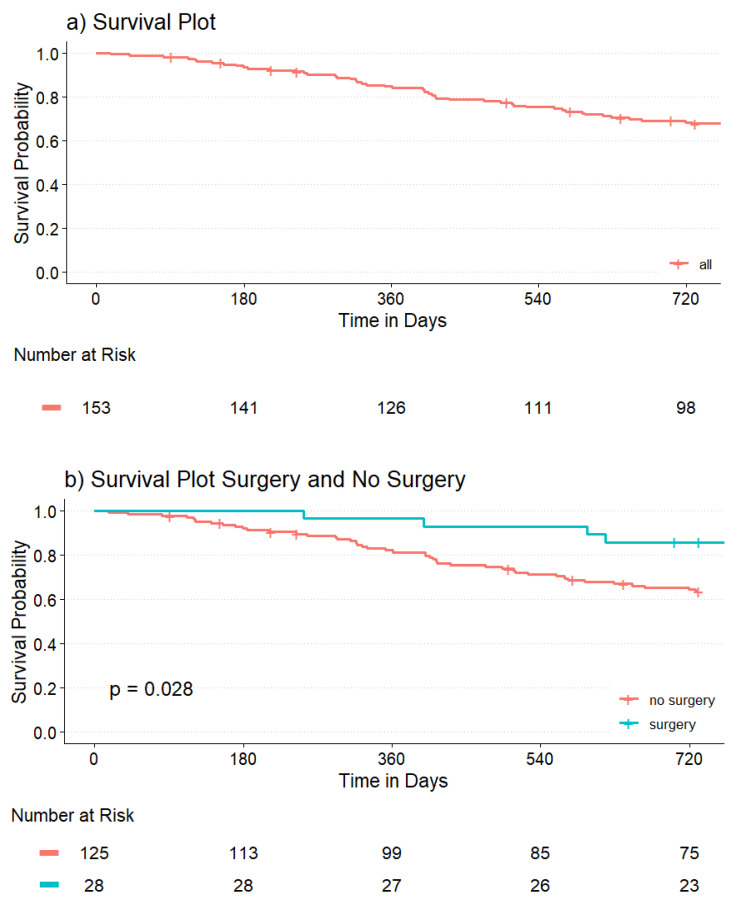
(**a**) Overall survival estimator and (**b**) survival estimator of patients grouped by surgery.

Patients who underwent VA-creating surgery had a better rate of survival after two years compared to patients who did not undergo access-creating surgery (86.2% vs. 63.3%; *p* = 0.028) (Figure 3).

There was a significant difference in the medians of time on KRT at the beginning of the study between the group that underwent ACF/G creation and the control group (median 1.16 years, Q1 1.16, Q3 4.17 vs. median 3.17 years, Q1 1.16, Q3 5.17, *p* = 0.013).

There was no significant difference in the Charlson Comorbidity Index between the group that underwent ACF/G creation and the control group (median 2 Q1 1, Q3 3 vs. median 3 Q1 1, Q3 4, *p* = 0.108). There was also no significant difference in median age between the two groups (median 65.5 years, Q1 57.5, Q3 72.75 vs. median 70.0 years, Q1 62.0, Q3 80.0, *p* = 0.069). The male-to-female ratio in the surgical group was 1:0.4; the ratio in the non-surgical group was 1:0.89. This difference in gender distribution was not statistically significant (*p* = 0.113).

The majority of deaths (68.8%) were attributed to cardiovascular events, infectious diseases other than CLABSIs, and malignancy. Three deaths (6.3%) were deemed access-related, with one patient who died during an access revision surgery, one patient who died from sepsis caused by a CVC-related bloodstream infection (CRBSI) and one patient who was declared “out-of-vessels” by their treating physicians and transferred to palliative care.

## 4. Discussion

Arteriovenous fistulas continue to be regarded as the most advantageous form of VA for most patients undergoing maintenance HD. The longstanding preference for AVFs is supported by their association with superior long-term patency rates, lower incidence of infectious complications and the potential for multiple future access sites [10,11]. However, emerging evidence suggests that the observed survival advantage in patients with functioning AVFs may be due not only to the technical and physiological benefits of the fistula itself, but also to the broader clinical context that enables AVFs to be created and used successfully.

In a large retrospective cohort study, which included 115,425 Medicaid patients aged 67 years or older initiating HD, outcomes were compared across three groups: those who initiated dialysis with a mature AVF, those who underwent an AVF creation attempt but began dialysis with a CVC, and those who started dialysis with a CVC without a prior AVF attempt. The 24-month mortality rates were 31% in the AVF group, 42% in the AVF CVC group, and 62% in the CVC-only group. Notably, even patients who began dialysis on a CVC but had undergone an AVF creation attempt demonstrated a survival benefit, suggesting that factors associated with the AVF creation process itself may contribute to improved outcomes. However, the authors’ risk factor analysis indicated that up to two-thirds of the observed survival advantage could be explained by patient-level characteristics present prior to the initiation of dialysis, such as overall health status, comorbidities, and access to predialysis care [7].

This and other studies raise the possibility that a substantial portion of the survival benefit associated with AVF use may, in fact, be driven by selection bias. That is, patients deemed suitable for AVF creation are often those with fewer comorbidities, better functional status, and earlier referral to nephrology care, factors that independently correlate with improved survival, regardless of access type.

To clarify the impact of access-related mortality, Quinn et al. conducted a retrospective cohort study that included 2300 patients who began their HD in five Canadian centers between 2004 and 2012. The study found that patients under the age of 65 who had a predialysis AVF creation attempt experienced significantly better long-term survival than those without an attempt. Interestingly, in patients over the age of 65, those who had undergone AVF attempts had superior survival during the first 24 months but worse outcomes beyond that period, raising questions about the long-term benefit of AVF creation in older, more comorbid populations. Importantly, only 2.3% of all recorded deaths in the study were attributed directly to access-related causes such as complications from access creation procedures or CVC-associated infections. This finding further emphasizes that broader clinical factors may be more critical to survival than access alone [12].

While selection bias and differences in patients’ comorbidities might partially explain the benefits seen in patients who underwent successful AVF creation and use, there is evidence that points to underlying healthcare management-specific issues and economic reasons.

Elements such as the duration of predialysis nephrology follow-up, timely referral for access creation, serum albumin and hemoglobin levels have all been associated with improved dialysis outcomes. Notably, these parameters tend to differ between patients who undergo AVF/G creation before dialysis initiation and those who do not, indicating disparities in access to and quality of predialysis care [13].

The proportion of people undergoing KTX as their KRT in Austria has historically been high. Overall, 48–49% of prevalent KRT patients had a functioning kidney transplant between 2019 and 2023. The high activity of KTX might have a moderate effect on CVC practice since CVCs could be used temporarily while waiting for a KTX. In Austria, however, CVC use steadily rose from below 20% in 2005 to 42.5% in 2023, while the rate of kidney transplantation activity decreased in the same period. Nevertheless, VA practice patterns and KTX activity are at least partially associated in multiple geographic regions [14,15].

Our findings from the Vienna ACTS NOW study add further context to this discussion. As previously reported, patients receiving HD at centers with in-house vascular surgery capabilities had significantly lower proportions of CVC use compared to those treated at centers without such access. The initial CVC proportions varied markedly across institutions, ranging from 27.6% to 94.4%, with an overall average of 42.5%. This disparity highlights the critical influence of institutional factors and infrastructure on VA practices.

All the centers participating in the study were publicly owned and financed through mandatory social health insurance. Six of the seven initially participating centers are affiliated with the same communal hospital operator. Each HD-providing unit is tasked with individually contacting VA-creating specialists, with no designated central VA-creating unit. This lack of streamlined access to care, combined with differences in in-house policies regarding access management and differing patient populations between centers, might explain the relatively low proportion of AVF/G creation attempts in our study population. Streamlined access to care also includes predialysis education by nephrologists with experience in VA management, enabling patients to make informed decisions on their preferred modality of KRT and raise awareness for the protection of potential access vessels. Missing coordination between healthcare providers could limit the options available to patients.

The newly collected two-year follow-up data reinforces this variability. Between 1 March 2023 and 1 March 2025, the overall rate of attempted conversions from CVC-based access to AVF or AVG was low, at just 18.3%. This conversion rate varied substantially between centers, with some reporting no VA creation attempts and others achieving a conversion rate as high as 40.9%. Of those who underwent AVF/G creation, 71.4% achieved successful cannulation, although this success was contingent on significant intervention: 18 patients required 12 surgical and 14 endovascular revisions to maintain or restore access function.

In our study population, patients undergoing VA-creating surgery had a better survival probability after two years than patients who did not (86.2% vs. 63.3%; *p* = 0.028). However, only three (6.3%) out of all deaths were directly related to VA management. Most deaths (68.8%) were attributed to cardiovascular events, infectious diseases other than CLABSIs, and malignancy. A significant portion of this difference in survival probability may be explained by selection bias, with healthier patients more likely to undergo VA-creating surgery.

Patients who consented to VA mapping were more likely to undergo VA-creating surgery than those who did not, with an odds ratio of 3.93. However, there was no significant difference in survival between the group that underwent mapping and those that did not.

Despite the technical and clinical challenges associated with AVF/G creation, contemporary data support the feasibility of performing these procedures in office-based or outpatient surgical settings. Even in cases requiring inpatient admission, hospital stays tend to be brief [16].

In our cohort, the median hospital admission for primary AVF/G creation was 3 days per patient (Q1 2 days, Q3 4 days), while revision procedures resulted in a median admission of 4 days per patient (Q1 2 days, Q3 11 days).

These extended periods of hospital admissions are also reflected in the reimbursement for VA creation. The Austrian MEL system allocates 2211 € for an AVF that is created in an outpatient setting or during a one-night admission. Higher amounts are allocated for longer admissions, with a diminishing amount for extended admissions. As no VA was created in an outpatient setting in our study population, the average reimbursement for initial AVF/G creation was 3130.44 € per patient. 

This effect is even stronger for surgical or endovascular revisions. A surgical revision using synthetic material is reimbursed with 3714.6 €, and an intervention on the upper extremity is reimbursed with 2809.8 € in an outpatient setting or during a one-night admission. Because of the extended admissions for patients undergoing surgical or endovascular revision, the average reimbursement was 11,893.02 € per patient.

The change in reimbursement is caused by the MEL system’s attempt to reflect the actual treatment cost and allocate more resources to the care of patients who require extended hospital admissions. Austrian federal states decide how to convert MEL points into a reimbursement in Euros for individual hospitals, with a conversion factor of 1:1 as a guideline. While a CVC implantation or change in a patient older than 74 years in an outpatient setting is reimbursed with 1939.8 €, after an admission of 7 days, the reimbursement is 9016 €. As elderly patients with infectious complications tend to need longer hospital admissions, the average reimbursement after a CVC-associated admission and CVC change was 8711.72 €. An admission caused by a CVC dysfunction was, on average, reimbursed with 6794.77 €. 

If a policy of outpatient-based AVF/G creation and service interventions in selected patients is adopted in Vienna, it could result in a significant reduction in hospital-admission days and total cost to the public healthcare system. The cost for hospital admissions triggered by CVC-related complications is unlikely to be easily modifiable.

There are several limitations to our study. All the centers that participated in the study are in the same city. The small sample size, especially in the subgroup that underwent AVF/G creation, may limit the generalization of our findings. This might also mask some of the expected differences between the group that underwent surgery and the one that did not, such as differences in age, sex or comorbidity. These undetected differences could mask potential selection bias, as patients selected to undergo access creation may differ clinically from those who remain on CVC-based hemodialysis.

Regarding the calculated costs, MEL-based reimbursements are a proxy for costs but ignore indirect expenses (e.g., lost productivity) or possible long-term savings from AVF/G usage.

Resource shortages and language barriers, as well as a long and possibly frustrating history of VA creation, could make it unlikely for individual patients to participate in a study examining the possibility of VA access creation.

However, we were able to show a continued variation in VA creation policies in Vienna, with the rate of attempted AVF/G creation in the study population ranging from 0% to 40.9% between centers over two years. A comprehensive VA management policy with a structured clinical pathway for vascular access management could alter the observed differences and ease patient access to healthcare resources.

## 5. Conclusions

Evidence from retrospective studies documents a survival benefit for patients undergoing predialysis AV fistula creation. There are also data on significant confounders and mediators influencing this association, such as patient-specific characteristics and the capability of the healthcare system to timely detect and treat CKD prior to the initiation of KRT.

The proportion of CVC- and AVF/G-based HD might therefore be associated with the level of access patients have to the healthcare system and the efficiency of the care network to identify and support patients in their runup to HD.

An answer to these open questions would provide physicians with evidence to help patients make informed decisions about their choice of VA access. It could also help to emphasize the importance of pre-HD care and screening programs for CKD.

Our own data point towards a difference in CVC use between different centers, not only driven by patient characteristics, but also by locally available resources and differences in VA management. Ultimately, “access to access” continues to be the primary determinant from an institutional perspective.

## Figures and Tables

**Table 1:** Sample characteristics.

	All (N = 191)	No AVF/G Surgery (n = 125)	AVF/G Surgery (n = 28)	Lost to Follow-Up (n = 38)
Demographics				
Male (% of total)	108 (56.5%)	66 (52.8%)	20 (71.4%)	22 (57.9%)
Female (% of total)	83 (43.5%)	59 (47.2%)	8 (28.6%)	16 (42.1%)
Male:female ratio	1:0.77	1:0.89	1:0.4	1:0.73
Age, years (median Q1, Q3)	70.0 (58.5, 80.0)	71.0 (62.0, 80.0)	65.5 (57.5, 72.75)	74.0 (58.0, 80.75)
Time on KRT, years (median Q1, Q3)	3.17 (1.16, 5.17)	3.17 (1.16, 5.17)	1.16 (1.16, 4.17)	2.16 (1.16, 4.92)
Comorbidity index, (median Q1, Q3)	3 (1, 3)	3 (1, 4)	2 (1, 3)	2 (1, 3)
VA mapping (% of total)	61 (31.9%)	39 (31.2%)	18 (64.3%)	4 (10.5%)
Mortality (% of total)				
Cardiovascular	14 (7.3%)	13 (10.4%)	1 (3.6%)	n/a
Infection	11 (5.8%)	10 (8.0%)	1 (3.6%)	n/a
Malignoma	8 (4.2%)	8 (6.4%)	0 (0%)	n/a
Access	3 (1.6%)	1 (0.1%)	2 (7.1%)	n/a
Other	5 (2.6%)	5 (4.0%)	0 (0%)	n/a
Unclear	7 (3.7%)	7 (5.6%)	0 (0%)	n/a
Total	48 (25.1%)	44 (35.2%)	4 (14.3%)	n/a
VA created				
Forearm AVF	12	0	12	n/a
Upper arm AVF	15	0	15	n/a
Upper arm graft	2	0	2	n/a
Lower extremity graft	1	0	1	n/a
Total	30	0	30	n/a
CVC changes				
Due to infectious complications	25	25	0	n/a
Due to CVC dysfunction	6	4	2	n/a
Due to other reasons	8	4	4	n/a
Total	39	33	6	n/a
CLABSI events	10	10	0	n/a
CRBSI events	11	8	3	n/a

**Table 2:** Center characteristics.

Center Code	Number of Patients on KRT in 2023	CVC Proportion at Initiation of the Study	Proportion of All Patients Included in the Study	Proportion of Included Patients who Underwent VA Mapping	Proportion of Attempted AVF/G Creation After 2 Years in Study Population
1	131	27.6%	86.5%	62.5%	18.8%
2	40	64.4%	72.4%	38.1%	0.0%
4	94	51.6%	45.8%	27.3%	40.9%
5	77	94.4%	58.8%	30.0%	10.0%
6	330	38.8%	30.3%	30.6%	11.1%
7	101	41.4%	90.2%	27.0%	18.9%

## Data Availability

The original contributions presented in the study are included in the article, further inquiries can be directed to the corresponding authors.

## Supplementary Materials

The following supporting information can be downloaded at https://www.mdpi.com/article/10.3390/jcm15072714/s1, Supplementary S1: Vienna ACTS NOW Study Group Acknowledgement for Collaborators.
