# Supplementary material for: Vascular Access Management in Patients on Catheter-Based Hemodialysis
Source: J Clin Med. 2026 Apr 3;15(7):2714. doi: 10.3390/jcm15072714 (PMC13073540; doi:10.3390/jcm15072714)
Supplement: Supplementary file 1 [file jcm-15-02714-s001.zip › jcm-4213182-supplementary.pdf]

Supplementary S1

Vienna ACTS NOW Study Group Acknowledgement for Collaborators

Department of Medicine VI, Clinic Ottakring, Vienna

Dr. Dr. Sara H. Ksiazek

Department of Medicine I, Clinic Landstraße, Vienna

Dr. Bruna Brunetta Gavranic

Dr. Eva Steineder

Wiener Dialysezentrum

Dr. Matthias Lorenz

Dr. Thomas Prikoszovich

Department of Medicine I, Clinic Favoriten, Vienna

Dr. Sabine Schmaldienst

Dr. Manfred Eigner

Department of Medicine III and Karl Landsteiner Institute for Metabolic Diseases and Nephrology, Clinic Hietzing, Vienna

Dr. Thomas Stulnig

Dr. Roland Edlinger

Department of Medicine III, Clinic Donaustadt, Vienna

Dr. Renate Klauser-Braun

Dr. Josef Kletzmayer
